# An Entropy-Based Clustering Algorithm for Real-Time High-Dimensional IoT Data Streams

**DOI:** 10.3390/s24227412

**Published:** 2024-11-20

**Authors:** Ibrahim Mutambik

**Affiliations:** Department of Information Science, College of Humanities and Social Sciences, King Saud University, P.O. Box 11451, Riyadh 4545, Saudi Arabia; imutambik@ksu.edu.sa

**Keywords:** Internet of Things (IoT), IoT data clustering, NSL-KDD dataset, memory consumption, sliding time window

## Abstract

The rapid growth of data streams, propelled by the proliferation of sensors and Internet of Things (IoT) devices, presents significant challenges for real-time clustering of high-dimensional data. Traditional clustering algorithms struggle with high dimensionality, memory and time constraints, and adapting to dynamically evolving data. Existing dimensionality reduction methods often neglect feature ranking, leading to suboptimal clustering performance. To address these issues, we introduce E-Stream, a novel entropy-based clustering algorithm for high-dimensional data streams. E-Stream performs real-time feature ranking based on entropy within a sliding time window to identify the most informative features, which are then utilized with the DenStream algorithm for efficient clustering. We evaluated E-Stream using the NSL-KDD dataset, comparing it against DenStream, CluStream, and MR-Stream. The evaluation metrics included the average F-Measure, Jaccard Index, Fowlkes–Mallows Index, Purity, and Rand Index. The results show that E-Stream outperformed the baseline algorithms in both clustering accuracy and computational efficiency while effectively reducing dimensionality. E-Stream also demonstrated significantly less memory consumption and fewer computational requirements, highlighting its suitability for real-time processing of high-dimensional data streams. Despite its strengths, E-Stream requires manual parameter adjustment and assumes a consistent number of active features, which may limit its adaptability to diverse datasets. Future work will focus on developing a fully autonomous, parameter-free version of the algorithm, incorporating mechanisms to handle missing features and improving the management of evolving clusters to enhance robustness and adaptability in dynamic IoT environments.

## 1. Introduction

The rapid proliferation of sensors in everyday life has led to an explosion in the volume of data streams. These streams, which consist of continuous sequences of data entries associated with integrated and precise timestamps, are expanding swiftly [1,2]. Numerous applications now generate vast and unpredictable volumes of data streams, primarily due to the growing presence of the Internet of Things (IoT), which connects real-time data sources. Efficient real-time analysis of these streams is crucial for obtaining valuable insights across various domains, including transportation, healthcare, social media, transaction logs, and internet activity [3,4]. Nevertheless, despite advancements in data analysis techniques, the exponential growth of data and its flow from diverse sources pose a significant challenge for analysts.

Clustering, one of the prominent methods for streamlining the analysis of data streams [5,6], has garnered considerable attention as the volume of data streams continues to increase [7]. Clustering aims to group data into meaningful clusters, facilitating better understanding and analysis. However, clustering algorithms face additional challenges when working with data streams, such as memory and time constraints, high dimensionality, and the necessity of processing data dynamically as it arrives [7,8].

Dimensionality reduction is closely linked to clustering in high-dimensional data streams, as it directly impacts clustering performance by addressing computational overhead, reducing noise, and improving the ability to identify meaningful patterns [8]. By transforming the data into a lower-dimensional space while retaining essential features, dimensionality reduction not only enhances computational efficiency but also increases the clustering accuracy. In dynamic data stream scenarios, dimensionality reduction is particularly crucial, as it ensures scalability and enables real-time analysis.

Three key challenges impact clustering in data streams, as follows: volume, velocity, and volatility [9,10,11]. The volume challenge requires algorithms to process large amounts of data rapidly. Velocity refers to the rapid rate at which new data are generated, while volatility represents a dynamic environment where evolving patterns constantly shift as data progresses [10,11].

As data continuously changes, algorithm structures and parameters must adapt in real time to handle new data streams. Clustering algorithms, thus, face challenges in adapting to shifting conditions that are not present in static datasets, particularly within rapidly evolving IoT environments [12,13]. Additionally, data streams often stem from unpredictable contexts, lacking prior knowledge of data distribution, which complicates the selection of the optimal parameters for outlier detection algorithms. Moreover, it is impractical to label the immense volume of data generated by the vast number of IoT devices in real-world scenarios, making unsupervised learning essential [7,13].

Numerous studies have focused on methods for dimensionality reduction within clustering algorithms, such as Hu [14] and Esfandiari [15]. However, these investigations largely overlooked the importance of feature ranking to pinpoint the most significant features. Feature ranking enhances dimensionality reduction by identifying and prioritizing the most relevant attributes, further improving the clustering performance in terms of both accuracy and resource efficiency. A more recent study by Ghosh [16] explored dimensionality reduction through genetic algorithms utilizing discrete wavelet transformation, single entropy, and spatial data. Nevertheless, this approach struggled to address continuously evolving data streams.

This leads to the following central research question: How can we develop an efficient, real-time feature selection method for clustering high-dimensional data streams that adapts to the dynamic nature of IoT environments?

In response, this paper introduces a novel entropy-based feature selection method called Entropy-Based Clustering for Data Streams (E-Stream). By integrating real-time feature ranking with dimensionality reduction, E-Stream improves the clustering efficiency and accuracy in high-dimensional data streams. E-Stream ranks features in real time based on their entropy, helping to identify the most essential feature subset. This subset is then applied to the DenStream algorithm for clustering, which optimizes memory usage and boosts system precision [17,18].

The remainder of this paper is structured as follows: Section 2 reviews the related works, including discussions on feature selection techniques and clustering algorithms for anomaly detection in data streams. Section 3 describes the methodology, providing a detailed explanation of the proposed algorithm along with its mathematical foundations, pseudocode, and a summary of the evaluation criteria and datasets used. Section 4 presents the results and analysis. Finally, Section 5 provides conclusions and suggestions for future work.

## 2. Review of Literature

With the expansion of IoT applications, an enormous volume of data is now being produced on a scale never before seen, driven by rapid technological advancements [19,20]. These data streams frequently involve multiple dimensions, posing challenges for both data processing and decision-making processes [21,22]. Richard Bellman was the first to coin the term curse of dimensionality, which pertains to the difficulty of managing high-dimensional datasets [23,24]. This challenge involves analyzing and managing datasets containing hundreds or even thousands of features, often derived from IoT sensors and devices. The abundance of features can introduce noise into the data streams, leading to increased complexity and model overfitting and diminished precision, along with higher computational demands for clustering techniques [25,26,27].

One of the primary challenges in clustering high-dimensional data streams lies in effectively evaluating these infinite streams of continuously evolving data while managing their storage for future analysis [28,29,30]. A key attribute of an efficient clustering method is its capacity to identify anomalies within such high-dimensional data streams [31,32,33,34]. In existing research, numerous algorithms have been introduced to tackle the problem of anomaly detection in these streams.

For instance, the MR-Stream algorithm [35,36] was developed to detect outliers. It functions by employing an online phase that stores condensed information on the evolving multi-density data as core mini-clusters, followed by an offline phase where a modified density-based clustering algorithm is applied to generate the final clusters. Furthermore, a grid-based approach is utilized to handle noise and variations in data densities. This algorithm has been comprehensively assessed using several quality metrics across both synthetic and real-world datasets, demonstrating notable improvements in scalability and clustering performance within multi-density data settings. However, MR-Stream struggles with computational overhead in high-dimensional spaces because of its reliance on grid structures, which can be inefficient when dealing with a large number of dimensions.

The Clustering Online Data Streams into Arbitrary Shapes (CODAS) algorithm is recognized as one of the pioneering approaches for online clustering [37,38]. It was developed to form clusters of various shapes using a stream-based clustering technique. CODAS works in real time, forming high-quality clusters from micro-clusters to summarize data points, and it can handle multidimensional data streams. Each micro-cluster is represented as a tuple *T* = (*N*,*C*,*R*,*E*), where *N* is the number of data points, *C* is the centroid, *R* is the radius, and *E* is the energy level. However, the clusters generated by the algorithm are static and do not evolve over time, limiting its applicability to dynamic data streams. Moreover, CODAS does not incorporate feature ranking or dimensionality reduction, which can lead to decreased efficiency in high-dimensional contexts.

To overcome this shortcoming, CODAS was advanced into a more sophisticated version, CluStream, which implements a two-phase mechanism to manage evolving data streams. This development marked the introduction of the first fully online clustering algorithm capable of handling such streams [39,40]. CluStream functions in the following two phases: first, micro-clusters are either formed or merged into existing ones. In the second phase, these micro-clusters are processed offline to form macro-clusters, which help define the final clusters. CluStream is efficient in generating high-quality clusters and can adapt to the evolving nature of data streams while also identifying noise. However, similar to other density-based clustering methods, CluStream demands significant processing time and does not effectively address the challenge of high dimensionality, as it lacks mechanisms for real-time feature ranking or dimensionality reduction.

An alternative improvement to CODAS has been proposed under the name i-CODAS, which emphasizes preserving distinct local radii for each micro-cluster [41,42]. In contrast to standard CODAS, similar to other density-based clustering methods, the radii for all micro-clusters are set and applied globally. Nonetheless, determining the optimal radius for micro-clusters is often difficult, and a single radius may not be appropriate for every micro-cluster. In reality, choosing an unsuitable radius can significantly reduce the clustering accuracy. The i-CODAS approach addresses this issue by dynamically adjusting each micro-cluster’s radius according to newly added data points, enabling real-time optimization of local radii. The data inside clusters is organized into micro-clusters, which are then represented in a clustering graph that shows their relationships. The final phase involves forming clusters of varying shapes based on this graph. Despite these benefits, i-CODAS faces the following key drawback: its substantial memory usage, particularly in high-dimensional data streams; it does not incorporate feature ranking or dimensionality reduction to mitigate this issue.

A completely online clustering approach based on density, known as DenStream, was later proposed [17,18]. DenStream continually adjusts the radii of micro-clusters to achieve optimal local values, effectively addressing a notable limitation of CluStream. It utilizes a two-tiered micro-cluster system, where less significant micro-clusters are stored temporarily, and an online pruning mechanism is employed to retrieve these clusters when needed. The experimental results show that DenStream provides a significant improvement compared to other clustering techniques. However, DenStream does not incorporate any form of feature ranking or dimensionality reduction, making it less efficient when dealing with high-dimensional data streams, as it may suffer from increased computational complexity and memory consumption.

To address these limitations, our proposed E-Stream algorithm builds upon DenStream by integrating real-time, entropy-based feature ranking within a sliding time window. This approach effectively reduces the dimensionality of the data stream by selecting the most informative features, thereby enhancing the computational efficiency and clustering accuracy. Unlike existing methods, E-Stream directly tackles the curse of dimensionality by reducing the number of dimensions in real time without compromising essential data characteristics.

A novel clustering technique for online data streams, named cluster-based efficient density grid method (CEDGM), was introduced by Tareq [43]. This approach leverages density grids for clustering with the primary objective of minimizing the number of distance function calculations while improving the overall precision of clusters. The algorithm operates in real time and is divided into two phases. In the first phase, core micro-clusters (CMCs) are generated, which are then merged to form macro-clusters during the second phase. This grid-based method has demonstrated effectiveness in reducing the number of distance computations and enhancing clustering outcomes, especially when applied to multi-density data such as sound datasets. Nevertheless, CEDGM exhibits a significant limitation, as it struggles to optimize memory usage, particularly in the presence of high-dimensional and sparse data. Additionally, it does not employ feature ranking or dimensionality reduction techniques to address high-dimensionality challenges.

In summary, while various clustering algorithms have been developed to process high-dimensional data streams, they often lack mechanisms for real-time feature ranking and dimensionality reduction, leading to inefficiencies in computational performance and memory usage. The proposed E-Stream algorithm addresses these gaps by incorporating entropy-based feature ranking, enabling efficient clustering of high-dimensional data streams with reduced computational overhead and improved accuracy.

## 3. Methodology

The E-Stream algorithm was created and implemented in Python. Its performance was subsequently evaluated against the following three widely used clustering algorithms: DenStream, CluStream, and MR-Stream. For the assessment, the NSL-KDD dataset [44,45], a well-established benchmark in clustering and anomaly detection for data streams, was used. This dataset, comprising over 4.8 million records of network traffic data, proved to be highly suitable for evaluating the algorithm’s effectiveness. Throughout the experimentation, anomalies were introduced to assess both the clustering accuracy and anomaly detection performance. The E-Stream algorithm’s performance was compared to the baseline methods across various tests. Each test was repeated five times with different subsets of data points, and the average results were calculated to determine the E-Stream algorithm’s overall efficiency.

### 3.1. E-Stream Algorithm

The E-Stream technique enhances the previous DenStream method. This new approach operates entirely in real time, specifically crafted to cluster high-dimensional data streams. It accomplishes this by utilizing feature ranking and ordering based on the information content found within the features. The process calculates entropy for selected features concerning a specific time window.

### 3.2. Overview of the E-Stream Algorithm

#### 3.2.1. Dimensionality Reduction

The goal of dimensionality reduction is to reduce the number of dimensions while preserving essential information. The algorithm utilizes feature ranking and ordering, driven by the informational value of the features. Entropy serves as a critical measure for assessing the information contained within the features. The entropy of specific features is calculated relative to the time window. As time progresses, varying weights are assigned to features based on their entropy over time—a concept known as temporal entropy—as explained in Equation (1).

(1) $$\overset{¯}{H}\left(x_{i},t_{1},t_{2}\right)=\frac{\sum_{t1}^{t2}\sum_{i=1}^{n}-p(x_{i,t})log\left[p\left(x_{i,t}\right)\right]}{t_{2}-t_{1}+1}\;$$

Determining the significance of various features over time requires establishing a specific time frame for analysis. This time frame, called a window, moves along the timeline and accumulates a sequence of data points. These points are then used to compute the entropy for each feature within the chosen window. Feature ranking plays a crucial role in determining the relative significance of features. The length of the time window is defined as $w=t_{2}-t_{1}+1$. At any given time t, the average temporal entropy is calculated over the defined time window. Consequently, the formula transitions from Equation (1) to Equation (2).

(2) $$\overset{¯}{H}\left(x_{i},t\right)=\frac{\sum_{t-w+1}^{t}\sum_{i=1}^{n}-p(x_{i,t})log[p\left(x_{i,t}\right)]}{w}$$

In order to enhance the model’s efficiency, a recursive approach is applied. Once the feature rankings are computed using temporal entropy, they are then ordered from the most important to the least.

#### 3.2.2. Reducing the Entropy Window

Within the selected time frame, entropy is calculated concerning the features, facilitating the reduction of high-dimensional data. The process begins when the incoming data stream is received. The main objective is to minimize the dimensionality of the dataset while preserving the essential information it contains. This is accomplished by assessing the entropy for each feature over the time window. As time progresses, different features are assigned varying levels of importance based on their computed entropy, a concept referred to as temporal entropy.

#### 3.2.3. Entropy-Based Clustering for Dynamic Data Streams (DenStream)

After completing the data reduction stage, the process moves into clustering the continuous stream of data and detecting any anomalies. In this step, calculating the window size is critical to ensuring it surpasses the speed of the data stream by starting a window counter. For each data stream in the dataset, the maximum ratio (Rmax) is first determined, and then the minimum ratio (Rmin) is calculated from Rmax. For every incoming data point, the corresponding micro-cluster is identified, and, if required, a new potential micro-cluster is generated. The procedure then updates the current micro-clusters and refreshes the energy levels of the core micro-clusters, while the energy for both potential and weak micro-clusters is updated separately. Finally, the macro-cluster graph is revised.

The focus here is on managing micro-clusters, which are small groups of data points that are continuously adjusted as new data are processed or old data discarded. These micro-clusters fall into the following three categories: potential, core, and weak.

- Potential micro-clusters are groups under evaluation to assess whether they fulfill the criteria to become core micro-clusters. These are still in an assessment phase and have not yet achieved core status;
- Core micro-clusters are those that have met the necessary criteria and are deemed significant, as they are held in buffer memory. These clusters provide critical insights and are prioritized for more detailed analysis, reflecting important data patterns;
- Weak micro-clusters refer to clusters that, due to the windowing mechanism, have lost their significance and no longer qualify as core micro-clusters. These clusters are gradually removed, allowing memory resources to be dedicated solely to the most relevant clusters.

In conclusion, this process is designed to dynamically refine micro-clusters as data evolves. By categorizing them into potential, core, and weak groups, the system ensures efficient management and storage of the most relevant clusters, enabling accurate and up-to-date analysis.

#### 3.2.4. Micro-Cluster Discovery

The initial phase of the search process consists of scanning a group of weak micro-clusters contained within the buffer using Equation (3). This filtering step is designed to eliminate irrelevant micro-clusters and concentrate on finding the micro-cluster pertinent to the task. If no suitable weak micro-cluster is identified, the algorithm restarts the search, trying to detect the target micro-cluster in the next set of micro-clusters. If the first two attempts are unsuccessful, the algorithm redirects its attention to the primary micro-cluster set in an effort to identify the core micro-cluster that fulfills the search parameters. In instances in which more than one micro-cluster meets the requirements of Equation (3), the algorithm selects one at random.

(3) $$d\left(X_{i},C\right)<R$$

#### 3.2.5. Micro-Cluster Modification

When a micro-cluster receives a new data point, its associated metadata are constantly updated. Should micro-cluster $T\;(Nt,\;N^{\prime }t,\;Ct,\;Rt,\;Et,\;ELt,\;and\;Mt)$ already be present at that moment and a new set of data points Xt+1 has been assigned to it, the metadata are refreshed in real time at t+1. The procedure for updating the local density Nt+1 includes an increment, as stated in Equation (4).

(4) $$\mathrm{Local}\;\mathrm{density},N_{t+1}=N_{t}+1$$

When T is either classified as a weak micro-cluster T ∈MC weak or a potential micro-cluster T ∈MC potential and its density surpasses the predetermined threshold Nt=The density, it transitions to the core group of micro-clusters (MC core). If T was previously part of, or has just been assigned to, a core micro-cluster (T ∈MC core), its radius (Rt+1) is repeatedly modified through the forgetting process outlined in Equation (5).

(5) $$\mathrm{Radius},R_{t+1}=min\left(\left[R_{t}+\left\{\frac{2\times d\left(X_{t+1},C_{t}\right)}{R_{t}}-1\right\}\times \frac{1}{\mathrm{Decay}}\right],R_{max}\right)$$

The radius of the micro-cluster is only adjusted when a data point is detected within the shell region, as points residing within the kernel region exert minimal effect on the radius expansion. In contrast, a point situated further from the center plays a larger role in this adjustment. When the data point Xt+1 is positioned within the shell, both the updated data count $N^{\prime }t+1$ and the new center of the micro-cluster Ct+1 are recalculated using Equations (6) and (8).

(6) $$N_{t+1}^{\prime }=N_{t}^{\prime }+1$$

(7) $$\mathrm{Center},C_{t+1}^{k}=\frac{\left(N_{t+1}^{\prime }-1\right)\times C_{t}^{k}+X_{t+1}^{k}}{N_{t+1}^{\prime }}$$

For dimensions k=1,2,3,…, D, where D denotes the number of dimensions of the data point, a new energy update function has been introduced. This function is aimed at continuously adjusting the energy of the micro-cluster Et+1. The energy accumulated by the micro-cluster is inversely related to the distance between the cluster center and the data points, demonstrating a well-balanced relationship. Equation (8) illustrates how the energy Et+1 of the newly identified core micro-cluster is updated. Bold changes reflect clarifications and wording adjustments.

(8) $$\mathrm{Energy},{\;E}_{t+1}=E_{t}+\left\{\frac{R_{t}-d\left(X_{t+1},C_{t}\right)}{R_{t}}\right\}\times \frac{1}{\mathrm{Decay}}$$

#### 3.2.6. Storing Weak Micro-Clusters in a Buffer

As the clustering process advances with each incoming data point, the energy levels of core micro-clusters progressively diminish, reflecting changes within the data stream. A core micro-cluster is considered inactive when its energy falls to zero or lower. Inactive micro-clusters are defined as those with energy levels less than or equal to zero E ≤0, meaning they are temporarily irrelevant. Once detected, these micro-clusters are transferred to buffer memory, and their energy is reset to half of the original value E=0.5. The inactive clusters are stored in this buffer for potential future use but do not engage in the active clustering graph, as any overlapping connections are broken.

#### 3.2.7. Elimination of Micro-Clusters

The energy allocated to each fading micro-cluster stored in the buffer is reduced by a decay factor, along with a reduction in the core micro-cluster’s energy (as described in Section 3.2.1). This approach helps to identify micro-clusters that have gradually weakened over time and are no longer relevant to the active data stream. A micro-cluster is deemed inactive if it is linked to zero or non-positive energy E ≤0, resulting in its permanent removal from the system’s memory.

## 4. Results

This section presents a comparative analysis of the E-Stream algorithm’s performance against DenStream, CluStream, and MR-Stream algorithms [17,18,21,22]. The configuration for the E-Stream algorithm included parameters such as a window size of 11, percentage of 0.88, decay rate of 50, micro-core threshold of 11, maximum radius of 0.1, and minimum radius of 0.05. For the other algorithms, the parameters are set with a decay rate of 50, a radius of 0.01, minimum threshold of 3, lambda set to 0.997, grid granularity at 10, minimum points of 3, and horizon of 2. To evaluate the clustering quality of the E-Stream algorithm after dimensionality reduction, an anomaly generation process was applied to the real-world NSL-KDD dataset. Subsequently, the runtime performance of E-Stream was benchmarked against DenStream, CluStream, and MR-Stream. The experiments were conducted five times, each with distinct random seeds. The NSL-KDD dataset comprises TCP connection logs from a LAN network, containing a total of 145,586 instances across 40 attributes.

### 4.1. Quality Evaluation

The quality of the E-Stream algorithm’s execution was assessed using the various metrics outlined below.

#### F-Measures

The F-Measure, often referred to as the F-Score, or F1 Score, combines precision and recall into a single metric. Precision is defined as the ratio of true positive predictions to the total number of positive predictions made, while recall measures the ratio of true positive predictions to the total number of actual positive instances. The F-Measure balances both precision and recall, with values ranging between 0 and 1, where 1 represents the best possible score and 0 the worst [21,22]. The formula for the F-Measure is given by the following:

(9) $$F-\mathrm{measure}\left(C,G\right)=\frac{2*precision\left(C,G\right)*Recall\left(C,G\right)}{precision\left(C,G\right)+Recall\left(C,G\right)}$$

**Figure 1:**
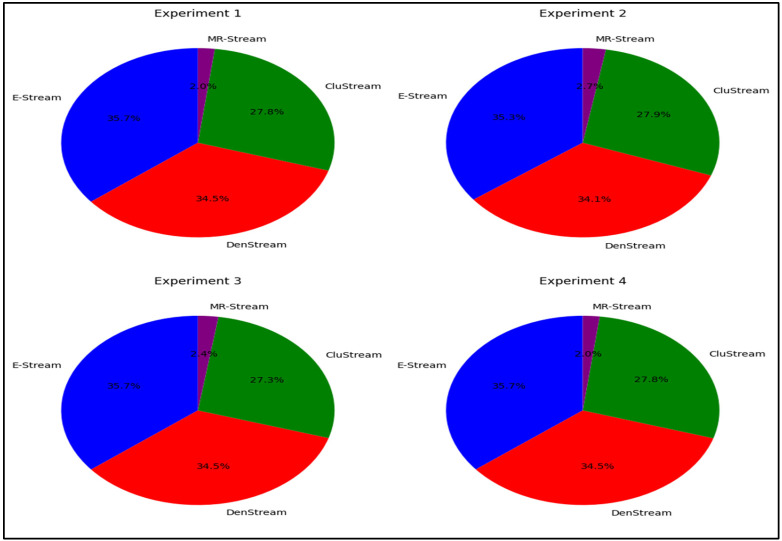
Comparative evaluation of E-Stream, DenStream, CluStream, and MR-Stream on the NSL-KDD dataset: F-Measure performance and dimensionality efficiency.

The comparison of the performances of the E-Stream, DenStream, CluStream, and MR-Stream algorithms on the NSL-KDD dataset, based on average F-Measure results, is depicted in Figure 1. The evaluation outcomes reveal that the E-Stream algorithm surpassed the other stream clustering algorithms (DenStream, CluStream, and MR-Stream) in terms of the F-Measures, with respective scores of 85%, 83%, 63%, and 4%. Despite E-Stream’s minor edge over DenStream, it still exhibited significant improvements compared to the CluStream and MR-Stream algorithms. Additionally, the E-Stream algorithm effectively reduced the data’s dimensionality, leading to fewer computations with memory overheads.

### 4.2. Jaccard Index (JI)

The Jaccard Index (JI) is a statistical method used to assess similarity and diversity among sample sets. It functions as an external assessment metric, as shown in several studies, such as Zhang et al. [21] and Osman et al. [22]. The formula for the Jaccard Index is as follows:

(10) $$Jaccard\left(C,G\right)=\sqrt{\frac{\left|TP\right|}{\left|TP\right|+\left|FN\right|+\left|FP\right|}}$$

where

- TP represents true positives;
- FP stands for false positives;
- FN refers to false negatives.

**Figure 2:**
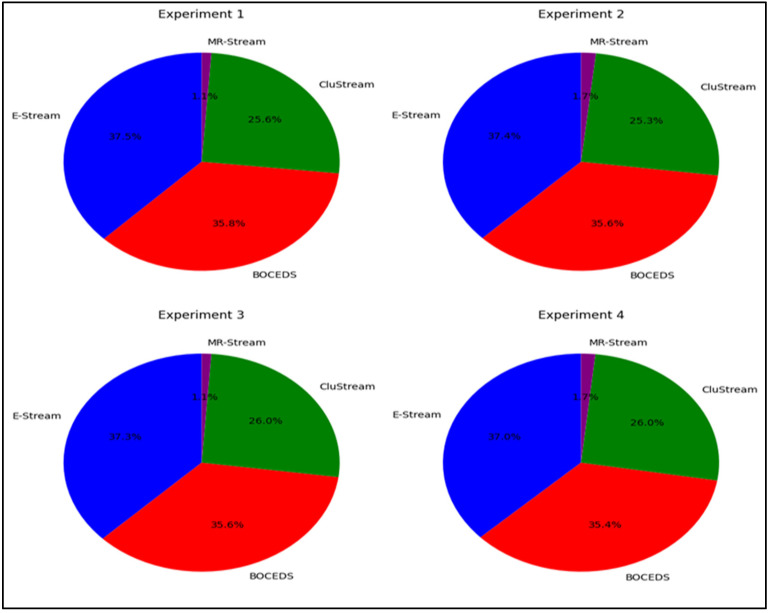
Performance of E-Stream, DenStream, CluStream, and MR-Stream on the NSL-KDD dataset in terms of the Jaccard Index.

The experimental results presented in Figure 2 compare the E-Stream, DenStream, CluStream, and MR-Stream algorithms using the Jaccard Index on the NSL-KDD dataset. E-Stream outperformed the baseline algorithms, achieving Jaccard Index values of 67%, 66%, 47%, and 6%, respectively. While the difference between E-Stream and DenStream in terms of the Jaccard score is minor, the E-Stream algorithm presents a substantial improvement when compared to CluStream and MR-Stream.

### 4.3. Fowlkes–Mallows Index (FM)

The Fowlkes–Mallows Index (FM) serves as a metric for assessing the resemblance between two sets of clusters. When the FM score is higher, this signifies a greater alignment between the clusters and the actual classification. This index is derived from the following formula:

(11) $$FM=\sqrt{\frac{\left|TP\right|}{\left|TP|+|FP\right|}\cdot \frac{\left|TP\right|}{\left|TP|+|FN\right|}}$$

**Figure 3:**
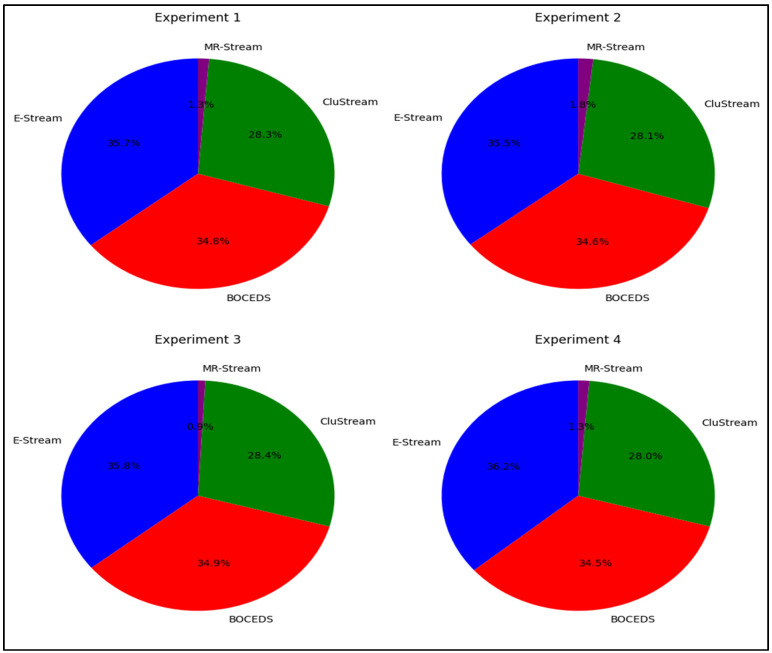
Fowlkes–Mallows Index comparison for E-Stream, DenStream, CluStream, and MR-Stream on the NSL-KDD dataset.

The experimental results, as illustrated in Figure 3, demonstrate that the E-Stream algorithm consistently outperformed the other algorithms (i.e., DenStream, CluStream, and MR-Stream) in all four experiments. E-Stream achieved the highest F-Measure (FM) scores, ranging from 35.5% to 36.2%, highlighting its robustness in clustering tasks. DenStream followed closely, with FM values between 34.5% and 34.9%, showing strong performances, albeit slightly behind E-Stream. CluStream exhibited moderate results, with FM scores around 28%, consistently lagging behind E-Stream and DenStream. MR-Stream performed the weakest, with FM values consistently below 2%, indicating its limited effectiveness compared to the other algorithms. These findings confirm that E-Stream is the most effective clustering algorithm across all experiments, as reflected in Figure 3.

#### 4.3.1. Purity

Purity is a measure utilized to evaluate the effectiveness of the E-STREAM algorithm. It is calculated by dividing the count of elements within each cluster by the total number of ground truth instances [46,47]. Purity scores vary from 0 to 1, with 0 indicating low purity and 1 indicating high purity.

(12) $$Purity=\frac{\sum_{i=1}^{N}n_{i}^{d}}{n_{i}}\;$$

**Figure 4:**
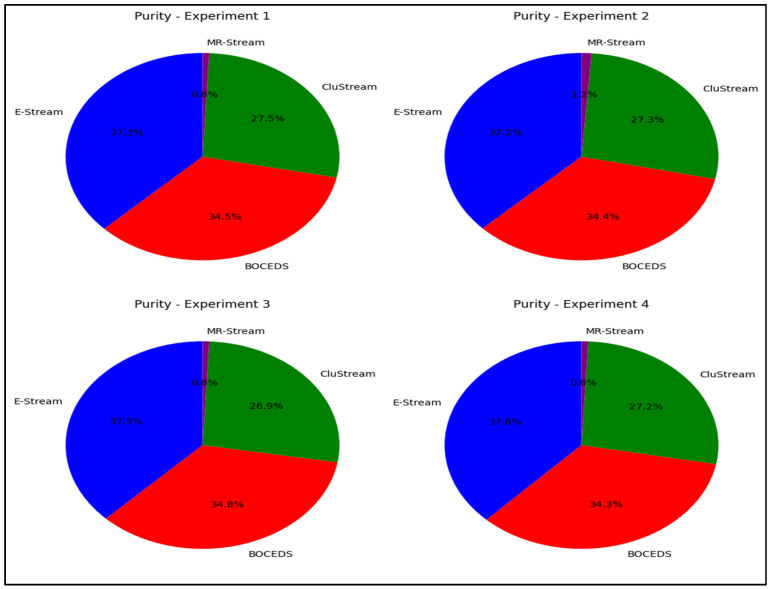
Comparative purity analysis for E-Stream, DenStream, CluStream, and MR-Stream on the NSL-KDD dataset.

The experimental results, as depicted in Figure 4, show that the E-Stream algorithm consistently outperformed the other algorithms—DenStream, CluStream, and MR-Stream—across all four experiments in terms of purity. E-Stream achieved the highest purity, with scores ranging from 37.2% to 37.8%, demonstrating its robust clustering accuracy. DenStream followed closely, with purity scores between 34.3% and 34.8%, showing strong but slightly weaker performances compared to E-Stream. CluStream exhibited moderate performances, with purity levels ranging from 26.9% to 27.5%. In contrast, MR-Stream consistently underperformed, with purity scores between 0.8% and 1.2%, indicating its limited effectiveness in aligning clusters with true labels. These findings clearly indicate that E-Stream is the most accurate algorithm in terms of purity, as reflected in Figure 4.

#### 4.3.2. Rand Index

The Rand Index is a statistical tool that measures how similar two different groupings are, specifically focusing on the level of agreement between the clustering results and the actual data labels [48,49]. This index is calculated by the following formula:

(13) $$\mathrm{Rand}\mathrm{Index}=\frac{\left|TP|+|TN\right|}{\left|TP|+|TN|+|FP|+|FN\right|}$$

**Figure 5:**
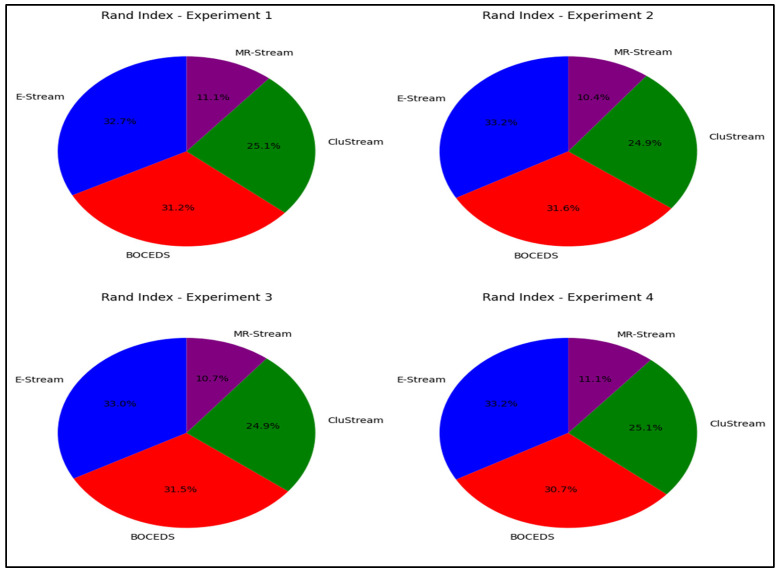
Rand Index comparison for E-Stream, DenStream, CluStream, and MR-Stream on the NSL-KDD dataset.

The experimental results, as shown in Figure 5, highlight the superior performance of the E-Stream algorithm, which consistently achieved the highest Rand Index (RI) scores across all four experiments. E-Stream’s Rand Index values ranged from 32.7% to 33.2%, indicating its strong ability to generate clusters that closely align with the actual data labels. DenStream followed closely behind, with Rand Index scores ranging from 30.7% to 31.6%, performing well but slightly below E-Stream. CluStream exhibited moderate performance, with Rand Index values between 24.9% and 25.1%. In contrast, MR-Stream consistently underperformed, with the lowest Rand Index scores ranging from 10.4% to 11.1%, indicating poor clustering accuracy. These results emphasize that E-Stream consistently produced the most accurate clusters, as demonstrated in Figure 5.

### 4.4. Complexity Assessment

**Figure 6:**
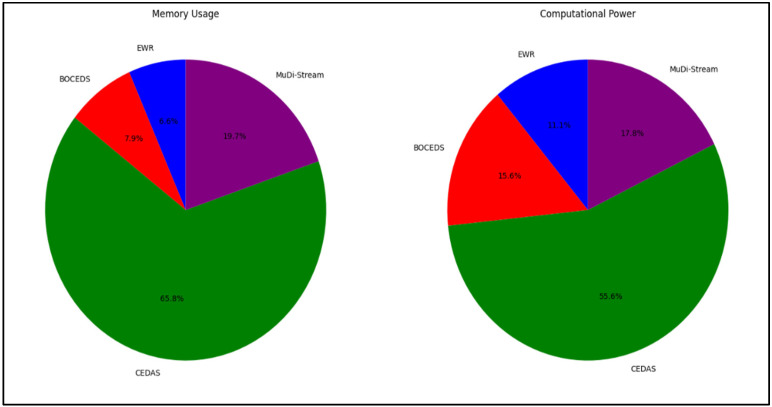
Memory usage and computational power comparison of E-Stream, DenStream, CluStream, and MR-Stream on the NSL-KDD dataset.

In the assessment of the computational complexity, the performance of the E-Stream algorithm was evaluated against the DenStream, CluStream, and MR-Stream algorithms, with an emphasis on memory consumption and computational efficiency. It is essential for stream clustering algorithms to ensure both minimal memory consumption and computational efficiency during their execution. Figure 6 presents the memory usage and processing efficiency of all tested algorithms on the NSL-KDD dataset.

As illustrated in Figure 6, the E-Stream algorithm exhibited comparatively lower memory consumption than the DenStream algorithm. This can be attributed to E-Stream’s use of a buffer to temporarily store data, and once the data points are no longer required, they are discarded to optimize memory usage. Notably, the E-Stream algorithm consumed substantially less memory than both CluStream and MR-Stream.

Moreover, a comparison of the computational efficiency between the E-Stream algorithm and the DenStream, CluStream, and MR-Stream algorithms is displayed in Figure 6. The figure demonstrates that the E-Stream algorithm requires significantly less computational resources compared to the other algorithms, with CluStream demanding the most computational power, while E-Stream exhibited the most efficient usage.

**Figure 7:**
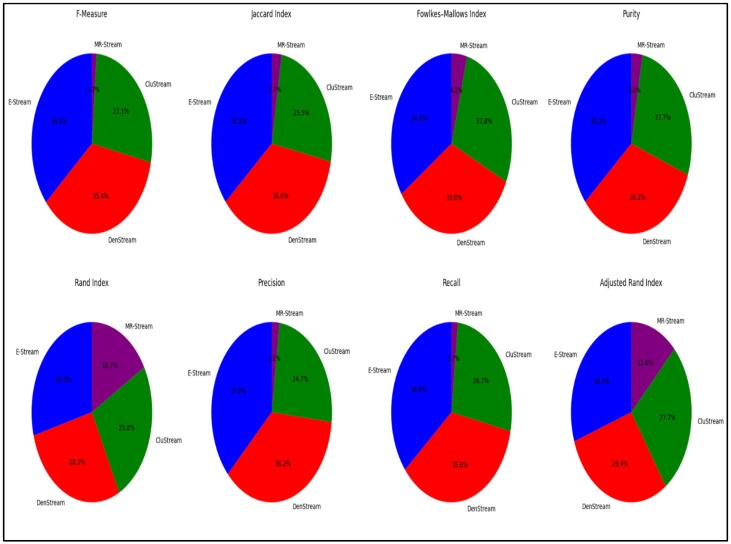
Breakdown of the evaluation metrics for E-Stream, DenStream, CluStream, and MR-Stream on the NSL-KDD dataset.

The experimental results consistently demonstrate that the E-Stream algorithm surpassed the other baseline stream clustering algorithms across a range of evaluation metrics. This strong performance can be attributed to its ability to reduce memory consumption and computational overhead, facilitated by its dimensionality reduction process. Additionally, the findings suggest that the E-Stream algorithm consistently produced high-purity results, even after reducing the dataset’s dimensions while still preserving key data features. Figure 7 summarizes the comparison of the E-Stream algorithm’s performance with DenStream, CluStream, and MR-Stream, using the NSL-KDD dataset across various evaluation metrics.

## 5. Conclusions and Discussion

This paper presented the E-Stream algorithm, an improvement over the previous DenStream algorithm, offering a fully online approach for clustering high-dimensional data streams. E-Stream utilizes feature prioritization and ordering based on the information content of features, achieved by calculating the entropy of selected features within a defined time window. The algorithm’s effectiveness in both dimensionality reduction and clustering accuracy was assessed through comparisons with other algorithms such as DenStream, CluStream, and MR-Stream. While our evaluation using the NSL-KDD dataset demonstrates the potential of E-Stream, we acknowledge that relying on a single dataset may limit the generalizability of our findings. These results demonstrate that E-Stream outperforms the baseline algorithms by effectively preserving essential data features while reducing dimensionality. Moreover, the E-Stream algorithm exhibited significantly lower memory consumption and computational requirements compared to DenStream, CluStream, and MR-Stream, further highlighting its efficiency in processing high-dimensional data streams.

Despite its advantages, the E-Stream algorithm has some limitations. It is not fully autonomous, requiring manual adjustment of specific parameters by the user. Implementing a fully autonomous, parameter-free algorithm would greatly enhance its adaptability across various datasets. Furthermore, the current algorithm assumes a consistent number of active features during execution, which ignores the possibility of missing or unavailable features. Since many datasets are likely to have absent features, incorporating mechanisms to address such scenarios is crucial for broader applicability. Another notable limitation is the lack of comparison with high-dimensionality reduction algorithms such as the Generalized Hebbian Algorithm (GHA) for online principal component analysis (PCA). Including such algorithms in our comparative analysis could provide deeper insights into the performance and scalability of E-Stream relative to established dimensionality reduction techniques. Additionally, the evaluation primarily compares E-Stream with core clustering algorithms, without direct comparison to existing feature ranking and selection methods for data streams. Including such comparisons would provide a more comprehensive assessment of the algorithm’s effectiveness in feature selection.

Moreover, the experiments were conducted using simulated datasets rather than real-world conditions. Conducting experiments in real experimental setups, such as deploying E-Stream on IoT hardware platforms, such as an ESP32 board with MQTT servers for real-time data transmission from IoT sensors, would provide practical insights into latency, scalability, and resource consumption under real-world IoT conditions. This would significantly strengthen the evaluation by bridging the gap between theoretical and applied performance.

Future work will focus on addressing challenges such as the incorrect merging of clusters, which occurs when overlapping moving clusters are erroneously combined. We also plan to benchmark E-Stream against established high-dimensionality reduction algorithms like GHA for online PCA to evaluate its performance in relation to these methods. Additionally, enhancing the management of evolving clusters that may adopt various shapes during execution is a priority. Furthermore, we aim to evaluate the algorithm on IoT hardware platforms and multiple datasets to provide a more comprehensive understanding of its real-world performance. Future work should also include a sensitivity analysis to evaluate the impact of variations in the configuration parameters on the algorithm’s overall performance. This will help identify optimal parameter settings and assess the robustness of the approach under diverse conditions. By tackling these issues, the E-Stream algorithm can be further refined to provide more robust and adaptable clustering solutions for high-dimensional data streams.

## Data Availability

Data can be made available upon request to ensure privacy re-strictions are upheld.
